# The Twilight Sleep Propaganda

**Published:** 1916-07-22

**Authors:** Alice M. Head

**Affiliations:** Editor *Woman at Home* and *Girl's Realm,* 20 Tavistock Street, Covent Garden, London, W.C.


*To the Editor of* The Hospital.

*To the Editor of* The Hospital.

### The Twilight Sleep Propaganda.

*To the Editor of* The Hospital.

Sir,—In your article on "Twilight Sleep" in the current issue the following sentence occurs: "We are far from any belief in the infallibility of medical experts, even when they are unanimous, as in this case they seem to be." If this sentence applies to the medical practitioners you have consulted on this question, of course no comment can be made, but if it applies to the profession in general throughout the country, may a lay person inquire in what estimation you hold the opinion of so famous an obstetrician as Sir Halliday Croom, who has more than once placed on record his absolute belief in the "Twilight Sleep" method of treatment, and according to his own statement has practised it for many years?

You accuse the author of the *Weekly Dispatch* articles of having suppressed the truth. Undoubtedly the impression left by your own article is that "Twilight Sleep" has no supporters to speak of among the medical profession in this country. This is contrary to fact, and it would only have been fair of you if you had given the names of those specialists who are practising the "Twilight Sleep" method of treatment with unqualified success, or, at least, had allowed their existence. I know that they do exist because I have had much correspondence with some of them in my efforts to discover the truth.—Yours truly, Alice M. Head, Editor

*Woman at Home* and *Girl's Realm*,
20 Tavistock Street, Covent Garden,
London, W.C., July 17, 1916.

[We suspect that the editor of *The Woman at Home* is using the word "specialist" in a somewhat different sense from that in which we used the term "expert." Our authorities, whom we called "medical experts," are one and all consultants in obstetric practice and either past or present members of the visiting staff of recognised hospitals in the front rank, and they are unanimous. We are aware that Sir Halliday Croom used to uphold the scopolamine-morphine method, and we take our correspondent's word that he still does so. Indeed, it was his paper published in 1909 which first directed our attention to the matter. Some medical men up and down the country use this method, of whom some at least have been drawn into it, against their better judgment, by popular clamour. Still, the fact remains that twilight sleep is dangerous to the babies, and that it actually kills a certain number of them. The action and procedure on this question of the Editor of *The Weekly Dispatch*, who has excluded material facts, can bring him no credit. They may, indeed, prove distinctly harmful to the reputation of this paper and of its proprietors.—Ed. The Hospital.]

